# Morphology, Age, and Growth of *Triplophysa strauchii* in Sayram Lake, Xinjiang, China

**DOI:** 10.3390/ani15071039

**Published:** 2025-04-03

**Authors:** Zhengwei Wang, Huimin Hao, Jie Wei, Hao Wu, Syeda Maira Hamid, Ruixian Lv, Huale Lu, Zhulan Nie

**Affiliations:** 1 College of Life Science and Technology, Tarim University, Alar 843300, China; 10757232145@stumail.taru.edu.cn (Z.W.); 10757223077@stumail.taru.edu.cn (H.H.); 120070006@taru.edu.cn (J.W.); 3041222201@stumail.taru.edu.cn (H.W.); 1107572024402@stumail.taru.edu.cn (S.M.H.); 8061222202@stumail.taru.edu.cn (R.L.); 10757241102@stumail.taru.edu.cn (H.L.); 2 Xinjiang Production & Construction Corps Key Laboratory of Protection and Utilization of Biological Resources in Tarim Basin, Alar 843300, China

**Keywords:** spotted thicklip loach, measurement traits, aging method, growth model

## Abstract

This study focused on *T. strauchii* in Sayram Lake, Xinjiang. In August 2023, a total of 768 samples were collected to investigate its morphological, age, and growth characteristics. *T. strauchii* has an elongated body with a slightly raised area behind the head. Its head is flat, the body is slender, the back contour is arc-shaped, the trunk is thick and round, and the tail is short. Principal component analysis (PCA) showed that the cumulative contribution rate of the first three principal components was 53.80%, which reflects the morphological characteristics of the species. Moreover, gender characteristics are not prominently manifested in external morphology. Discriminant analysis showed an accuracy rate of 51.80%, indicating that the accuracy of gender discrimination relying solely on external morphology is limited. The species’ age distribution ranges between 1 and 7 years old, with the dominant age around 3 years old, and age structure showing a unimodal distribution. The relationship between body length and body weight is *W* = 7.432 × 10^−6^ *L*^3.037^(*R*^2^ = 0.995, *n* = 768). The exponent 3.037 indicates a growth pattern with priority given to body mass growth because it is greater than 3. The von Bertalanffy growth equation was selected to describe the growth of *T. strauchii*. The body length growth equation is *L_t_* =139.346 [1 − *e*^−0.267(*t*+1.639)^], and the body mass growth equation is *W_t_* = 27.79 [1 − *e*^−0.267(*t*+1.639)^]^3.073^. The inflection point age (*t_i_*) is 2.563, the growth coefficient (*k*) is 0.267, and the growth characteristic index(*φ*) is 3.715. The growth rate decreases with age, and the growth inflection point ages of males and females differ. The research findings provide basic data for population assessment, resource protection, and rational fishing in fishery resource management. This highlights the ecological adaptability of *T. strauchii* and emphasizes the importance of comprehensively considering multiple factors in fishery management.

## Simple Summary

In August 2023, we studied 768 samples of *T. strauchii* in Sayram Lake, Xinjiang, with the goal of exploring its morphology, age, and growth. *T. strauchii* has an elongated body with specific features. PCA showed that the first three principal components’ cumulative contribution rate was 53.80% for its morphology, and gender discrimination by external morphology had a 51.80% accuracy rate. Fish age was mainly 3 years old. The length–weight relationship indicated allometric growth. The von Bertalanffy equation described its growth with specific parameters. Growth rate decreased with age and differed between genders. These findings offer basic data for fishery management, highlighting the fish’s adaptability and the need to consider multiple factors in management, benefiting population assessment, resource protection, and rational fishing.

## 1. Introduction

Sayram Lake, located within the alpine basin in the western segment of the Tianshan Mountain Range, lies to the southwest of Bole City in the Bortala Mongolian Autonomous Prefecture, Xinjiang Uygur Autonomous Region. Its geographical coordinates range from 81°05′ E to 81°15′ E in longitude and 44°30′ N to 44°42′ N in latitude, with the elevation of the lake surface reaching up to 2073 m [1]. As the preeminent alpine cold-water lake in Xinjiang, it assumes a critically significant role within the regional ecosystem [2]. The ecosystem of Sayram Lake involves an intricate and intimate interaction among diverse terrestrial ecological types, including snow-capped mountains, glaciers, forests, and grasslands, as well as the lacustrine aquatic ecosystem. This gives rise to a complex and intricate ecological network [3]. Serving as a water-conserving source in the mountain range, Sayram Lake not only serves as a habitat for a multitude of rare and endangered flora and fauna, but also fulfills a fundamental role in modulating the climate of northern Xinjiang, significantly contributing to substantial upholding of the ecological equilibrium and stability across the region [4].

*T. strauchii* (Kessler, 1874) belongs to Cypriniformes, Cobitidae, Nemacheilinae, and the *Triplophysa* genus. It is locally known as “pike fish”. It is widely distributed in the rivers and lakes along the northern side of the Tianshan Mountains in Central Asia [5]. This species plays dual roles as both a predator and prey; it feeds on small invertebrates and affects their population [6], and serves as a food source for larger predators [7]. Notably, *T. strauchii* is an indicator species of ecosystem health and is closely related to fishery protection. It is highly sensitive to changes in water quality and habitat, such as pollution, alterations in water flow, and the destruction of vegetation. A decline in its population indicates potential threats to the fishery ecosystem [8,9,10].

Regarding the exploration of the biological features of *T. strauchii*, studies on morphology and age growth are of crucial importance. Fish morphology is the outcome of long-term environmental adaptation and is closely correlated with survival, behavior, and ecological functions [11,12,13]. For example, body shape influences hydrodynamic drag and concealment [14], while fin shape is related to swimming and maneuverability [15]. The age determination of fish commonly relies on calcified structures such as scales, otoliths, and opercula [16]. Among them, otoliths are preferred for the precise age determination of fish due to their stable chemical composition and distinct growth increments [17]. Additionally, the lapillus exhibits more advantages in age determination in comparison to the sagitta and asteriscus [18]. The research on the age growth of *T. strauchii* extends beyond simply determining age; it also requires the consideration of growth rate, the relationship between body length and body weight, etc. By collecting data on body length and body weight from different age groups and employing scientific methods, the growth pattern can be dissected and significant parameters, such as asymptotic body length and growth coefficient, can be determined [19,20]. This research is of great significance for understanding the growth mode and population dynamics of *T. strauchii*.

However, while studies on morphology, age, and growth are crucial for understanding fish biology, research on *Triplophysa* has centered on distribution [21], taxonomy [22], phylogeny [23], and genetics [24]. However, there is limited understanding of how its morphology, age, and growth interact with the broader ecosystem, especially regarding population resilience and competition with introduced species. Thus, our main goal is to fill this gap by conducting in-depth research on the age and growth of *T. strauchii*. By analyzing growth patterns and related parameters, we aim to contribute to the evaluation of its population resilience, improving our understanding of competitive dynamics and supporting the formulation of better conservation and management strategies.

## 2. Materials and Methods

### 2.1. Sample Collection and Processing

**Figure 1:**
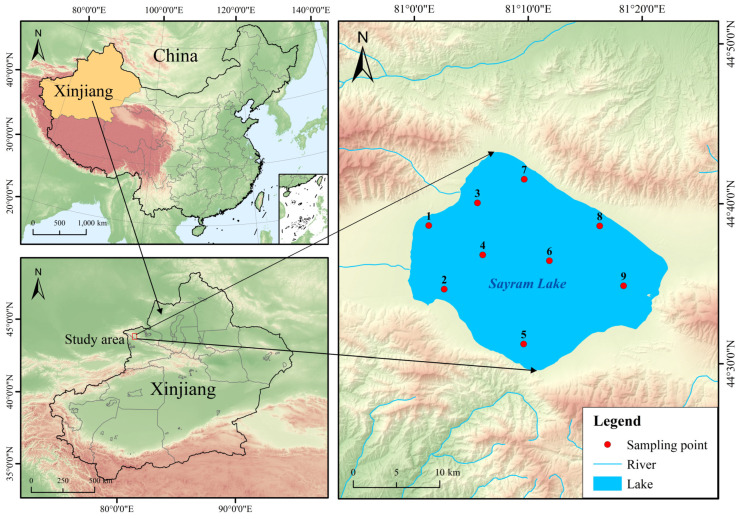
Diagram of sampling points.

In August 2023, fishing using multiple methods simultaneously was carried out in Sayram Lake (Figure 1). Specifically, we employed three-layer gillnets (with an inner mesh size of 5.5 cm and an outer mesh size of 18 cm) and traps (with a mesh size of 1 cm). A total of 768 *T. strauchii* specimens were collected (318 females and 413 males). Immediately after collection, the samples were anesthetized using 35 mg/L MS-222 (Fujian Jinjiang Aquatic Products Co., Ltd., Jinjiang, China) anesthetic [25]. Subsequently, routine biological measurements were conducted. The measurement precision for body length was 1 mm, and the body weight was accurate to 0.1 g. Once the measurements were completed, the samples were fixed and preserved with 10% formalin (Fuchen Chemical Reagent Co., Ltd., Tianjin, China) and taken back to the laboratory for further processing [26]. All data were collated, analyzed, and graphed using Excel 2016, SPSS 27.0, and Origin 2022.

### 2.2. Biological Determination

#### 2.2.1. Traditional Morphological Measurement

**Figure 2:**
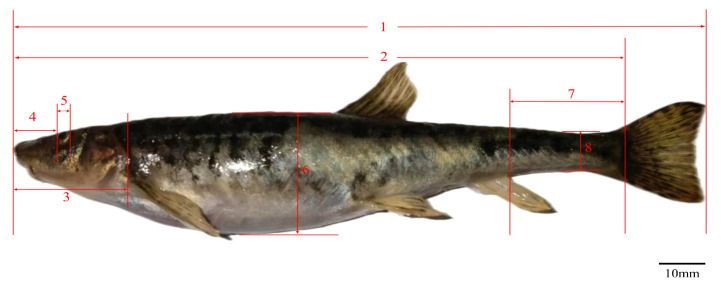
Morphological measurement of *T. strauchii*—1: total length; 2: body length; 3: head length; 4: snout length; 5: eye diameter; 6: body depth; 7: caudal peduncle length; 8: caudal peduncle height.

The measurable trait measurement indices of *T. strauchii* (Figure 2) mainly include total length, body length, body depth, head length, snout length, eye diameter, caudal peduncle length, and caudal peduncle depth, among others.

#### 2.2.2. Truss Morphometry Measurement

**Figure 3:**
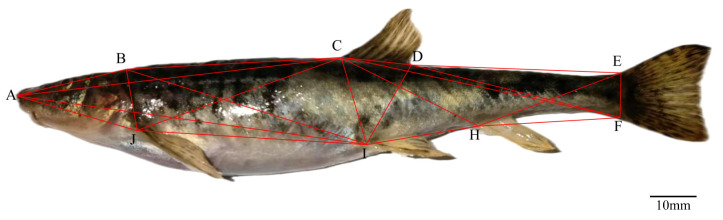
Truss points and distances for truss morphometry of *T. strauchii*—A: tip of snout; B: the last end of the frontal maxilla; C: origin of dorsal fin; D: basal end of the dorsal fin; E: dorsal origin of caudal fin; F: ventral origin of the caudal fin; H: anal fin origin; I: ventral fin origin; J: pectoral fin origin.

For truss morphometry, coordinate points were selected based on the method proposed by [27] and labeled as A, B, C, D, E, F, H, I, and J. Connecting these points yielded 21 truss distances (Figure 3), such as A–B, B–C, and so on. For example, A–B represents the distance from the snout tip to the back of the head, B–C from the back of the head to the start of the dorsal fin, and so forth.

### 2.3. Sex Determination

The sex of all *T. strauchii* was determined by naked-eye inspection after dissection. Individuals of undetermined sex were classified as juveniles (*n* = 37). Female *T. strauchii* show distinct ovarian changes: starting small, nearly transparent, and smooth during oogonial proliferation, then enlarging, turning light yellow with surface granules in the primary oocyte growth stage, darkening with visible large oocytes at maturation, growing with plump and glossy oocytes in the secondary oocyte stage, and reaching their maximum size, golden and with closely arranged eggs, when fully mature. In contrast, male *T. strauchii* undergo testicular development: testes are small, pale pink or milky white, smooth, and homogeneous in the spermatogonial proliferation stage; they expand, turn milky white and develop surface texture in the primary spermatocyte growth stage, grow larger, become harder with clearer textures and granules at maturation, continue to expand with a milky white color, hard texture, and dense structure in the secondary spermatocyte stage, and reach their peak size (milky white, hard, and smooth) during sperm formation [28].

### 2.4. Lapillus Processing and Age Estimation

**Figure 4:**
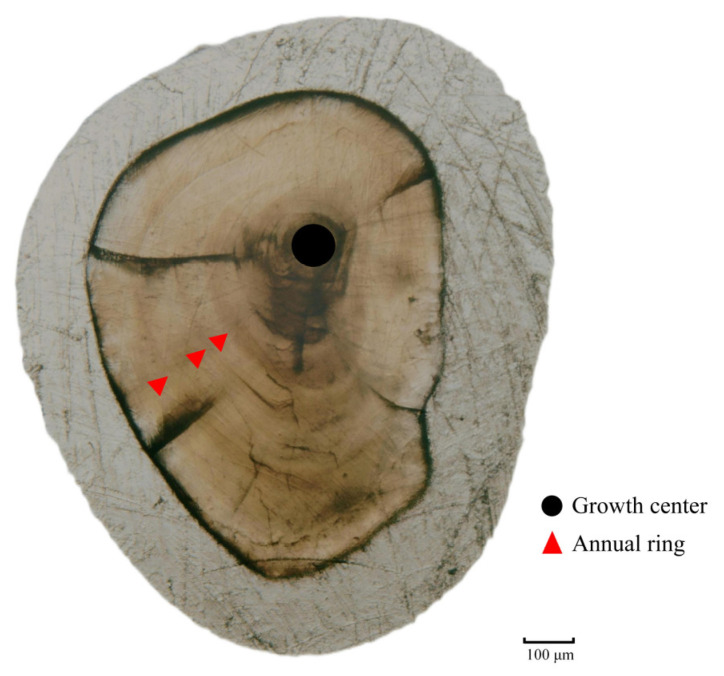
Growth zones of *T. strauchii*.

The age of *T. strauchii* was determined using the lapillus [29]. The specific operation procedures were as follows: during the dissection process, the lapillus was carefully taken out and placed into a 0.5 mL centrifuge tube. Then, 95% alcohol was added, and the tubes were properly marked [30]. After 1 to 2 days, the lapillus was taken out from the centrifuge tube, placed on a glass slide, and fixed with transparent nail polish. Once the lapillus was fixed firmly, it was ground carefully with sandpaper ranging from 3000# to 5000# until the growth center was reached. When the nucleus area of the lapillus could be clearly observed under a microscope, the method of blind inspection by 4 people was adopted to carefully observe the annual ring characteristics and then estimate age [31]. For age estimation of the lapillus, the method of identifying and counting annual rings is as follows: for 1-year-old individuals, it is the stage from the first annual ring not yet fully formed to the one just formed; for 2-year-old individuals, it is the stage from the formation of a new ring outside the first annual ring to the second annual ring that is just formed, and so on (Figure 4).

### 2.5. Relationship Between Body Length and Body Weight

The relationship between body length and body weight was fitted by a power function model, as shown in Equation (1).

*W* = *aL^b^* (1)

The condition factor was calculated by the Fulton method, as presented in Equation (2).

*K* = 100 *W/L*^3^ (2)

Note: *W*: body weight; *L*: body length; *a*: the growth condition factor; *b*: the allometric growth factor.

### 2.6. Growth Equation

An in-depth analysis of the growth relationships for the female population (*n* = 318), the male population (*n* = 413), and the overall population (*n* = 768) of *T. strauchii* was carried out using the von Bertalanffy growth equation [32].

The standard formula for the von Bertalanffy growth equation is as follows:

*L_t_* = *L_∞_*(1 − *e*^−*k*(*t*−*t*^_0_^)^) (3)

*Wt* = *W_∞_*(1 − *e^−k^*^(*t*−*t*^_0_^)^) (4)

Growth rate equation:

*dL/dt* = *L_∞_ke^−k^*^(*t*−*t*^_0_^)^ (5)

*dW/dt* = *bW_∞_ke*^−*k*(*t*−*t*^_0_^)^(1 − *e*^−*k*(*t*−*t*^_0_^)^)*^b^*^−1^ (6)

Growth acceleration equation:

*d*^2^*L/dt*^2^ = −*kL_∞_e^−^*^(*t*−*t*^_0_^)^ (7)

*d*^2^*W/dt*^2^ = *bW_∞_k*^2^*e*^−*k*(*t*−*t*^_0_^)^(1 − *e*^−*k*(*t*−*t*^_0_^)^)*^b^*^−2^(*be*^−*k*(*t*−*t*^_0_^)^ − 1) (8)

Growth inflection point age equation:

*t_i_* = *t*_0_ + *lnb/k* (9)

Growth characteristic index (*φ*) equation:

*φ* = *logk* + 2*logL_∞_* (10)

Note: *L_t_*: body length (mm) at age *t*; *W_t_*: body weight (g) at age *t*; *L*_∞_: asymptotic body length (mm); *W*_∞_: asymptotic body weight (g); *k*: growth coefficient; *t*_0_: assumed theoretical starting age of growth; *dL/dt*: body length growth rate; *dW/dt*: body weight growth rate; *d*^2^*L/dt*^2^: body length growth acceleration; *d*^2^*W/dt*^2^: body weight growth acceleration; *b*: index of the relationship between body length and body weight.

## 3. Results

### 3.1. Traditional Morphology

#### 3.1.1. Morphological Description

*T. strauchii* has an elongated body, a slightly raised back of the head, an arc-shaped dorsal profile, a thick and round anterior body, and a short tail. The caudal peduncle length was more than 3.5 times the caudal peduncle height. The head was slightly flattened, and the head width was slightly greater than the head height. The snout was obtuse; the mouth was inferior. The upper lip margin had many papillae, with 1 row at the anterior margin and 2–3 rows at the corners of the mouth. The lower lip surface was thick, with many deep folds. The middle groove was relatively deep with one papilla on each side of the isthmus. The lower jaw was spoon-shaped and did not protrude outside the lip. There were three pairs of barbels: the end rostral barbel reached the corner of the mouth, the side rostral barbel reached the anterior margin of the eye or the lower margin of the eye, and the mental barbel reached or exceeded the posterior margin of the eye. The anterior and posterior nostrils were only separated by skin protrusion and were in front of the eye. The eye was in the middle of the head and was laterally superior. There were no scales with smooth skin and complete lateral lines. The back and both sides were yellowish brown or grayish brown, with irregular small black spots, while the abdomen was light yellow. The dorsal fin and caudal fin had small markings, and the other fins were relatively bright. The dorsal fin was located slightly behind the center of the body with a slightly concaved margin. The base of the unbranched fin rays became hardened. The pectoral fin was fan-shaped and positioned laterally at the thorax. The fin counts were as follows: dorsal fin III, 7–8; anal fin III, 5; pectoral fin I, 7–8. The inner side of the gill rakers of the first gill arch was 12–14.

#### 3.1.2. Measurable Traits

Among the 10 measurable morphological traits of *T. strauchii*, body depth (13.66 ± 3.57 mm) was slightly greater than body width (11.85 ± 3.22 mm), and caudal peduncle length (21.57 ± 4.95 mm) was much greater than caudal peduncle height (6.43 ± 1.82 mm). The standard deviation of total length and body length, which measured 22.68 and 19.07, respectively, was relatively large. In contrast, the standard deviations of the other traits were relatively small. This indicated that *T. strauchii* has different body sizes and a wide size coverage range (Table 1).

To reduce the influence of individual specifications on the experiment, the ratios between the measurable shapes of males and females were calculated. Judging from the standard deviation of the trait ratios, the variation ranges of total length/body length and body depth/body width of male and female individuals were the smallest, while the variation range of body length/eye diameter was the largest (Table 2).

#### 3.1.3. Principal Component Analysis

Excluding the *T. strauchii* individuals whose genders could not be identified, a PCA was carried out on the ratios of 13 measurable traits. The contribution rates of the first principal component (PC1), the second principal component (PC2), and the third principal component (PC3) were 23.61%, 17.18%, and 13.00% respectively, with the cumulative contribution rate reaching 53.80% (Table 3).

**Figure 5:**
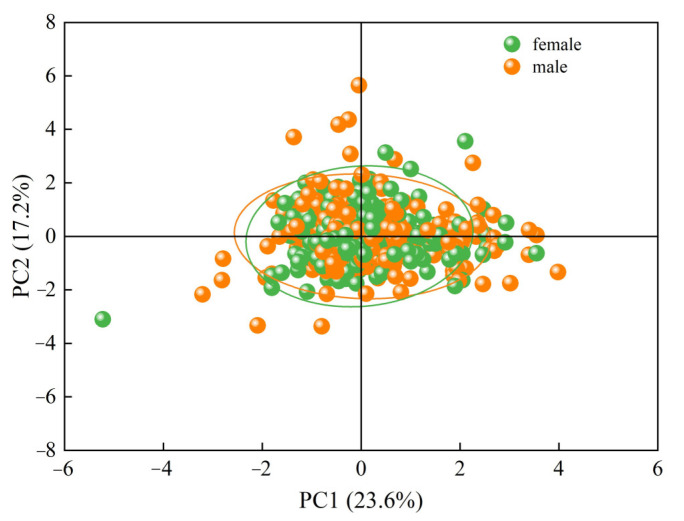
Distribution of males and females in PC1 and PC2.

In PC1, the trait ratio that plays a dominant role is head length/eye diameter. In PC2, the dominant traits mainly include head length/caudal peduncle length and body length/caudal peduncle length. In PC3, the dominant traits mainly include body height/body width and body length/body width. In the constructed two-dimensional scatter plot (Figure 5), the male and female individuals of *T. strauchii* considerably overlap, indicating that it is rather difficult to determine the sex of *T. strauchii* merely based on external morphological characteristics.

#### 3.1.4. Discriminant Analysis

The discriminant analysis method was utilized to analyze the ratio parameters of 13 measurable traits of female and male *T. strauchii* (*n* = 731). Based on this analysis, a corresponding discriminant function system was established. The specific function expressions are as follows:

For the female population:

Y1 = 279.811X_1_ − 84.405X_2_ + 97.474X_3_ + 636.413X_4_ + 6.994X_5_ − 31.877X_6_ − 0.439X_7_ + 12.21X_8_ − 3.681X_9_ + 3.268X_10_ + 228.526X_11_−44.561X_12_ + 13.663X_13_ − 639.987

For the male population:

Y2 = 279.780X_1_ − 84.068X_2_ + 97.160X_3_ + 634.105X_4_ + 6.980X_5_ − 30.916X_6_ − 0.564X_7_ + 12.198X_8_ − 3.528X_9_ + 3.829X_10_ + 224.123X_11_ − 44.485X_12_ + 13.783X_13_ − 638.534

In the formula, X_1_–X_13_ represent the ratios of total length/body length, body length/body width, body length/body height, body height/body width, body length/head length, body length/caudal peduncle length, body length/eye diameter, body length/interorbital distance, head length/snout length, head length/eye diameter, head length/caudal peduncle length, and caudal peduncle length/caudal peduncle height, respectively. Our results show the comprehensive discriminant accuracy for male and female *T. strauchii* is 51.80%.

### 3.2. Truss Structure

**Figure 6:**
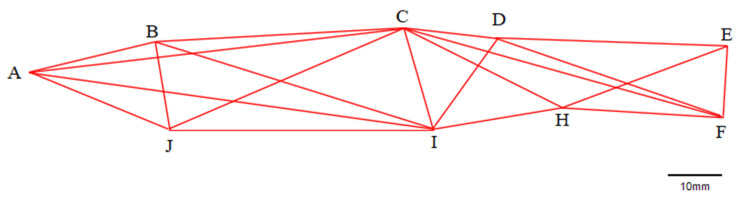
Truss structure of *T. strauchii*. A: tip of snout; B: the last end of the frontal maxilla; C: origin of dorsal fin; D: basal end of the dorsal fin; E: dorsal origin of caudal fin; F: ventral origin of the caudal fin; H: anal fin origin; I: ventral fin origin; J: pectoral fin origin.

The linear distances of the nine adjacent coordinate points of *T. strauchii* were standardized (Table 4). Subsequently, based on Table 4, a truss structure diagram of *T. strauchii* was constructed. It can be observed that *T. strauchii* exhibits the characteristics of a flat head and an elongated body (Figure 6).

### 3.3. Age Estimation

**Figure 7:**
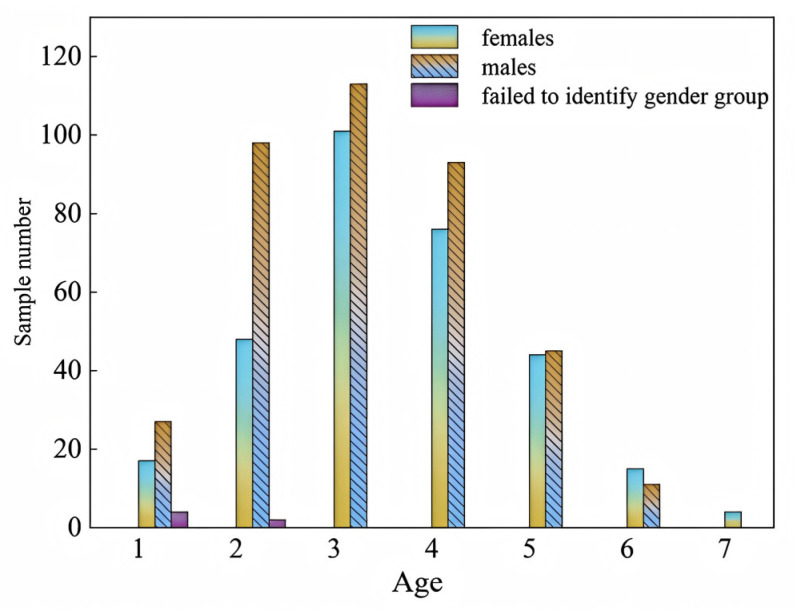
The age makeup of *T. strauchii* catch.

The age of 698 *T. strauchii* specimens from which the lapilli had been removed was estimated. The age distribution of *T. strauchii* mainly ranged from 1 to 7 years old. The dominant age group of the female population was 3 to 4 years old, and that of the male population was 2 to 3 years old. The proportion of older individuals of *T. strauchii* in the catch was relatively low. Individuals aged 6 to 7 years old accounted for 4.29% of the total sample size. Additionally, the dominant ages were mostly concentrated around 3 years old (Figure 7), indicating that the age structure of this population is relatively simple and tends to be younger.

### 3.4. Population Structure Characteristics

**Figure 8:**
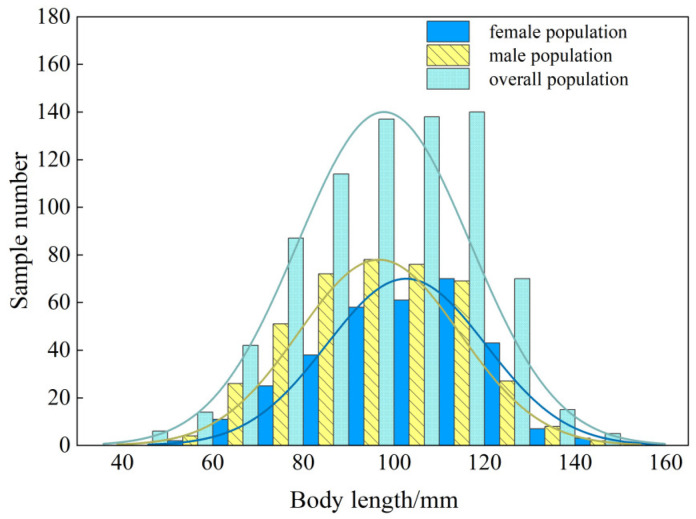
The body length distribution of *T. strauchii*.

The body length of *T. strauchii* (*n* = 768) ranged from 43.50 to 142.14 mm, with an average value of 97.79 ± 19.07 mm. For the female population (*n* = 318), the body length ranged from 52.14 to 142.14 mm, with an average value of 102.70 ± 17.52 mm. The body length range of the male population (*n* = 413) was 55.15 to 142.12 mm, with an average value of 96.70 ± 17.70 mm (Figure 8).

**Figure 9:**
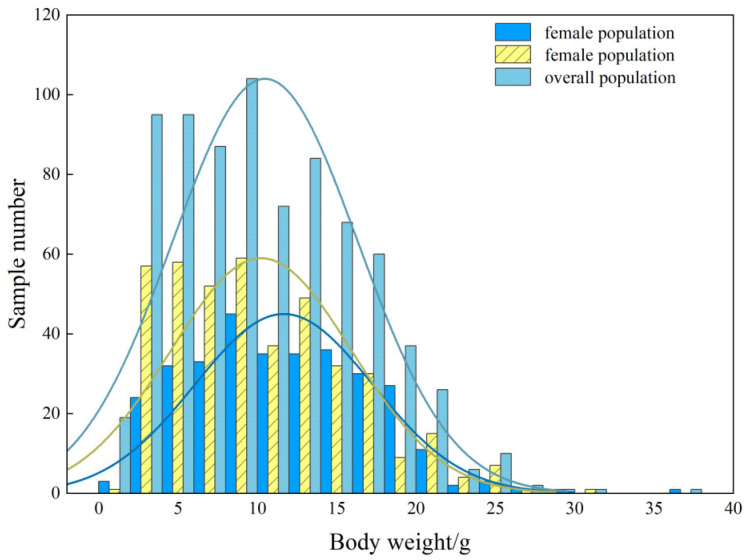
The body weight distribution of *T. strauchii*.

The weight of *T. strauchii* ranged from 0.60 to 36.71 g, with an average value of 10.49 ± 5.85 g. The weight range of the female group was 1.81–36.71 g, with an average value of 11.67 ± 5.61 g, while for the male population it ranged from 1.80 to 31.72 g, with an average value of 11.26 ± 5.70 g (Figure 9). The difference in weight between the male and female populations was also extremely significant (*p* < 0.01).

### 3.5. Body Length–Weight Relationship

**Figure 10:**
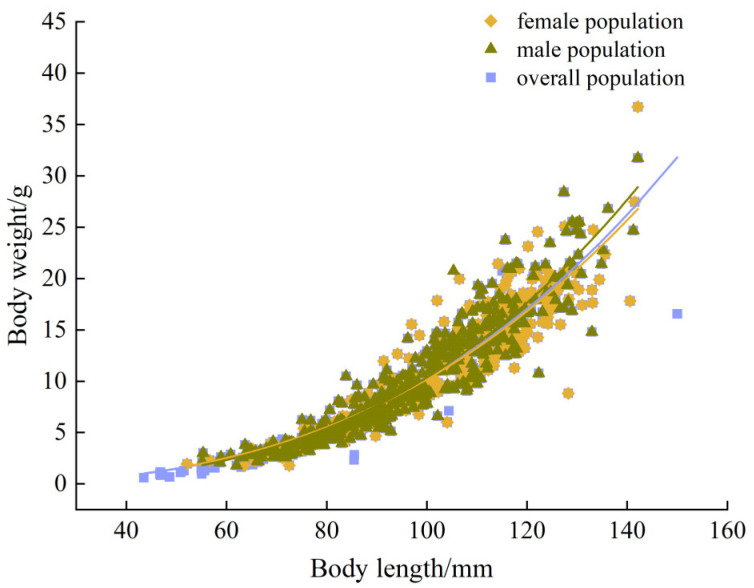
The length–weight relationship of *T. strauchii*.

The power function *W* = *aL^b^* was used to conduct detailed fitting of the correlation between the body length and body weight of the overall population of *T. strauchii*, as well as the female and male populations, respectively (Figure 10). *T. strauchii* showed an allometric growth pattern, with priority given to body mass growth (*b* > 3).

For the female population, the following equation was used:

*W* = 1.219 × 10^−5^
*L*^2.961^ (*R*^2^ = 0.988, *n* = 318), *b* = 2.961.

For the male population, the following equation was used:

*W* = 6.614 × 10^−6^
*L*^3.105^ (*R*^2^ = 0.992, *n* = 413), *b* = 3.105.

For the overall population, the following equation was used:

*W* = 7.432 × 10^−6^
*L*^3.073^ (*R*^2^ = 0.995, *n* = 768), *b* = 3.073.

### 3.6. Degree of Fatness

**Figure 11:**
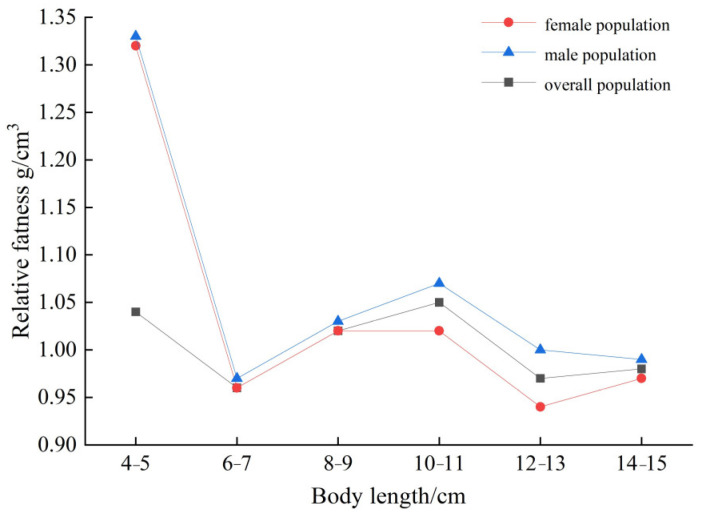
The changes in plumpness of *T. strauchii* of different body lengths.

The degree of fatness of *T. strauchii* in Xinjiang was studied by dividing body length into 2 cm intervals. The results indicated that, apart from the 4–5 cm length interval in which the fatness degree of both female and male *T. strauchii* exceeded 1.10 g/cm^3^, the fatness degree in every other length interval was at most 1.10 g/cm^3^ (Figure 11). In general, the degree of fatness of male *T. strauchii* was slightly higher than their female counterparts.

### 3.7. Growth Characteristics

**Figure 12:**
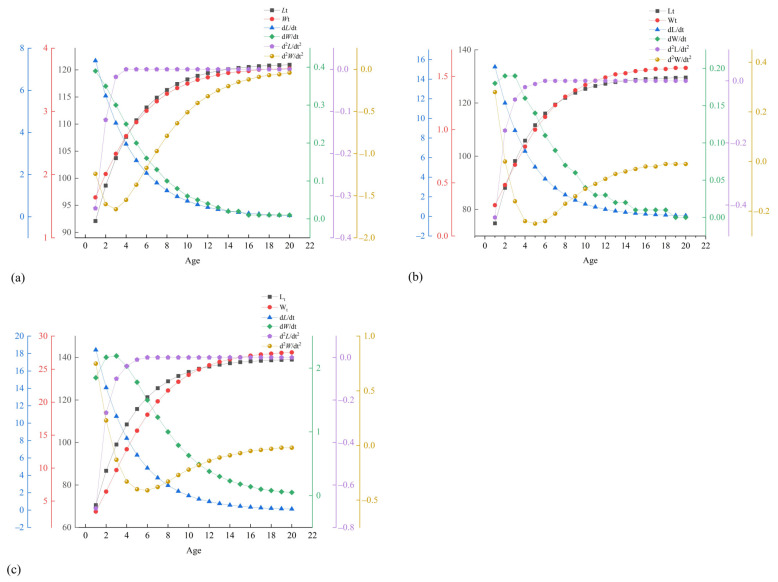
Growth-related curves of body length and body weight of *T. strauchii*. (**a**) Growth-related curves of female population; (**b**) growth-related curves of male population; (**c**) growth-related curves of overall population.

The von Bertalanffy growth equations and their derivatives showed a decline in growth rate with age, with inflection points at 2.30 years for females and 1.99 years for males (Appendix A). Both the female and male populations of *T. strauchii* manifested analogous patterns in the growth trends of body length and weight, as well as in the growth rate and acceleration of body length. As age advanced, the growth rate curve of body length exhibited a downward propensity, whereas the growth rate curve of weight attained its apex at the inflection point age. Upon surpassing the inflection point age, the growth rate of weight progressively decelerated and ultimately approached zero. Prior to reaching the age of 4, the growth acceleration curve of body length increased abruptly, and the growth acceleration curve of weight decreased steeply. However, after the age of 4, the growth acceleration curve of body length gradually assumed a downward trajectory and ultimately converged to zero, while the growth acceleration curve of weight gradually adopted an increased inclination and ultimately leveled off at zero (Figure 12).

## 4. Discussion

### 4.1. Individual Morphological Characteristics

The body of *T. strauchii* is elongated, with a slightly raised area behind the head, a rounded back contour, a thick and rounded front part, and a short tail. This body structure demonstrates significant adaptability for survival in Sayram Lake. Its streamlined body shape reduces swimming resistance in the water flow, enabling it to better adapt to the lake’s water flow environment [33]. This is consistent with the findings that the streamlined body of wild *Danio rerio* (Order Cypriniformes, Family Cyprinidae) is beneficial for fast and persistent swimming in a uniform and rapid water flow [34]. The slightly flattened head and inferior mouth position are convenient for foraging at the bottom of the lake [35]. The characteristic of having no scales on the body surface and a complete lateral line is beneficial for its survival in a low-temperature and low-oxygen water body environment. The lateral line system enables *T. strauchii* to sensitively perceive changes in water flow and surrounding environmental information, thus aiding in avoiding predators and searching for food resources in a timely manner [36]. In terms of morphology and physiology, regarding the adaptive characteristics of scaleless fish to low-temperature and low-oxygen environments, the integrity of the lateral line system is one of the key factors [37]. The lateral line system plays a crucial role in fish’s perception of environmental change, as well as their predation and defense behaviors, and scaleless body surfaces may reduce heat loss in low-temperature environments, as observed in the survival strategy of *T. orientalis* [38]. These morphological characteristics collectively indicate that *T. strauchii* has evolved a combination of morphological characteristics well suited to the ecological environment of Sayram Lake, reflecting the long-term evolution process. This is an important manifestation of its adaptation to the survival environment.

In this study, PCA was used to analyze the ratios of 13 measurable traits of male and female individuals of *T. strauchii*. The cumulative contribution rate of the first three principal components reached 53.80%, mainly concentrated in the head, body shape, and tail. In PC1, the ratio of head length to eye diameter dominates, and this ratio reflects the coordination between the visual organs and the development of the entire head, as well as the ecological adaptation features. This ratio is closely related to behaviors such as foraging [39] and avoiding predators [40]. In a deep-water area with relatively dark light or a complex lake-bottom environment, an appropriate ratio of head length to eye diameter helps *T. strauchii* to detect prey and potential dangers more efficiently. In PC2, the ratios of head length to caudal peduncle length and body length to caudal peduncle length have an important impact on its movement ability, growth, development, and body proportion coordination. Caudal peduncle length plays a key role in swimming and turning flexibility [41]. In a turbulent water flow area, a longer caudal peduncle makes it easier for the fish to control direction and maintain stability; in a relatively calm water area, slight differences in body proportions have an impact on its growth rate and energy allocation strategy. In PC3, the ratio of body height to body width and body length to body width determines body shape and space utilization strategies, and affects the morphological adaptability of *T. strauchii* in different aquatic environments. These traits have an impact on the layout and function of the internal organs, ensuring the normal operation of digestive, reproductive, and other systems [42]. This adaptive change in body proportion helps *T. strauchii* to occupy a specific ecological niche in the Sayram Lake ecosystem, differentiating it from other species in terms of resource utilization and living space. All this will maintain the diversity and stability of the lake ecosystem.

Discriminant analysis is widely used in differentiating population differences [43]. In this study, discriminant analysis was carried out on the ratio parameters of 13 measurable traits of male and female *T. strauchii*. The results showed that the accuracy rate of gender discrimination was 51.8%. In contrast to these previous studies, in previous studies on the morphology and growth of two *Culter* species (Order Cypriniformes, Family Cyprinidae), a discriminant equation with nine characteristic values was established, achieving a 100% accuracy rate [44]. After analyzing the population variables of *Harpadon nehereus* (Order Aulopiformes, Family Synodontidae), the comprehensive discriminant rate was 88.8% [45]. In contrast, the difference in external characteristics between male and female *T. strauchii* was not obvious, resulting in a relatively low discriminant analysis accuracy rate. These results indicate that in practical applications, relying solely on these external morphological features for gender discrimination has significant limitations. To improve accuracy, other methods such as gene analysis techniques or the further exploration of more discriminative morphological features should be considered.

### 4.2. Age and Growth Features

The reproduction and dynamics of a population are closely linked to its age structure and growth characteristics. Age and growth are key indicators in the study of fish biology and important parameters for predicting changes in fishery resources [46]. The age of *T. strauchii* caught from Sayram Lake was mainly concentrated at 3 years old, with a relatively small proportion of older fish. Compared with *T. marmorata* in the Maerkang River [47], *T. strauchii* exhibited smaller inflection age and shorter rapid growth period. Moreover, the inflection age of females was found to be significantly greater than males. The ovarian development in females requires the accumulation of a large amount of nutrients to produce eggs, leading to the allocation of more energy to gonadal development in the initial growth stage. This delay in the growth process of other parts of the body results in a relatively later growth inflection age for females [48]. In the study of *Ptychobarbus dipogon* (Order Cypriniformes, Family Cyprinidae), another species in the plateau area [49], a similar trend was observed, where the inflection age of females was greater than that of males.

The introduction of *Coregonus peled* (Order Salmoniformes, Family Salmonidae) in 1998 led to intensified competition for food resources, the compression of living space, and other problems [50]. *T. strauchii* may adjust its growth strategy and enter the stages of sexual maturity and reproduction ahead of time to ensure the continuation of its population. As pointed out in relevant research such as [51,52], when species are confronted with survival pressures, they often change their growth and reproductive patterns to adapt to environmental changes. However, this adaptation puts the species at a disadvantage in terms of competition with other species. When competing for limited food resources, the slower growth rate results in a relatively weak ability to acquire food. This age structure and growth pattern are the result of long-term natural selection. Under specific environmental conditions, smaller individuals and a shorter growth cycle are beneficial for improving the survival probability and reproductive success rate of the population [53].

When performing a nonlinear fitting of the body length and weight of fish using the equation W = *aL^b^*, the power index b is usually used to assess the growth state of fish [54]. Overall, *T. strauchii* showed an allometric growth pattern, with priority given to body mass growth (*b* > 3). The increase in body mass signifies the accumulation of more fat and other energy reserves, enhancing its adaptability to the harsh environment at higher altitudes [55]. Similarly, another study on *Poecilia mexicana* (Order Cyprinodontiformes, Family Poeciliidae) revealed that reductions in body size led to a significant decrease in the energy requirements of all populations living in extreme environments [56]. These findings strongly corroborate that the optimization of body mass and the rational allocation of energy reserves are key strategies for enhancing survival ability in a challenging harsh environment.

The results of fitting the von Bertalanffy model showed that the apparent growth index *φ* is positively correlated with growth rate [57]. The apparent growth index of *T. strauchii* is relatively larger than that of *T. stewarti* [32], indicating faster growth. According to the classification standard of fish growth rate [58], fish with a *k* value between 0.20 and 0.50 are fast-growing fish. This result is closely related to the unique ecological conditions of Sayram Lake. The water in Sayram Lake is rich in dissolved oxygen, providing sufficient oxygen supply for the respiratory metabolism of *T. strauchii* and supporting its high energy demand. This is consistent with the research findings on the impact of dissolved oxygen on the growth of *Ictalurus punctatus* (Order Siluriformes, Family Ictaluridae) [59]. At the same time, there are many algae and much organic debris in the lake, which serve as diverse food sources and nutritional elements that meet the nutritional needs of *T. strauchii* at different growth stages, thus providing a solid material basis for its rapid growth.

The population of *T. strauchii* at the sampling site of Sayram Lake exhibited a unique distribution pattern in terms of body length and weight. When compared with other congeneric fish (Table 5), its body length and weight were significantly lower than those of *T. yarkandensis* inhabiting the Yarkant River. This finding is consistent with previous research in the field of fish growth and environmental relationships [60]. Fish of the same genus in different river ecosystems often display distinct growth differentiations due to significant habitat differences [61]. The population of *T. strauchii* in Sayram Lake is larger in both body length and weight than that of *T. stewarti* in Chugutso, Tibet. This indicates a gradient change in the abundance and quality of food resources and overall environmental conditions across different lake ecosystems. These changes have a varying impact on the growth of fish of the same genus [62]. Despite inhabiting different rivers, there exists a certain degree of similarity in some ecological conditions, which gives rise to a convergent evolutionary trait. For instance, traits such as body length range and other morphological characteristics enable fish to better adapt to the environment. This aligns well with the view proposed earlier in the study of fish ecological adaptability [63].

The condition factor is an index for evaluating the plumpness and nutritional status of fish, reflecting their fatness and growth situation [69]. Previous research has shown that the condition factor decreases with increases in body length, a finding consistent with prior conclusions [70]. Generally, female fish, which need to carry eggs, tend to have a higher condition factor than that of males [71]. However, in this study, the fish were caught in August, just after the breeding season. The spawning of females led to a decrease in their condition factor. The energy required for the development of eggs and spawning to ensure the progress of reproduction depletes large amounts of energy reserves, resulting in a reduced body energy reserves and a lower condition factor [72]. In contrast, males do not experience this high-energy-consuming spawning process and allocate most of their energy primarily for maintenance and growth. Their energy reserves are further utilized for courtship, enhancing courtship competitiveness and reproductive success rates [73]. During the growth and reproduction process, it is necessary to balance the allocation of energy in different physiological processes to better adapt to environmental conditions and ensure the continuation of the population. Therefore, a sufficient food supply before the breeding period is a key factor to improve the reproductive capacity of the population, increase the survival rate of juvenile fish, and promote the healthy development of the population [74].

## 5. Conclusions

This study focuses on *T. strauchii*, a species highly adapted to Sayram Lake. Its streamlined form, which is an adaptation to the aquatic environment, along with specialized head–mouth features and a scaleless body with an intact lateral line, reduces water resistance, aids foraging, and enhances environmental awareness. Evolved body proportions help it carve out a unique ecological niche and maintain ecosystem stability. However, it is vulnerable. It has a relatively simple age structure, struggles with food competition, and needs much energy for breeding. Moreover, it has indistinct sexual dimorphism, complicating population studies. Environmental differences also set the growth traits of *T. strauchii* apart from those of congeneric fish. To safeguard this species, pollution-causing human activities in nearby lakes should be curtailed, pre-breeding food supply should be secured, and fishing should be regulated. Future research can use molecular biology for gender distinction, aiding holistic conservation strategies to maintain the lake’s biodiversity and ecological stability.

## Figures and Tables

**Table 1:** Measurable characteristics of *T. strauchii*. (*n* = 768; mm).

Traits	Range	Mean ± SD ^1^
Total length	51.35~185.77	116.50 ± 22.68
Body length	43.50~142.14	97.79 ± 19.07
Body width	3.74~23.43	11.85 ± 3.22
Body depth	3.83~24.33	13.66 ± 3.57
Head length	3.40~30.13	19.68 ± 3.92
Snout length	1.44~13.84	7.81 ± 2.10
Eye diameter	0.93~6.29	3.17 ± 0.75
Eye spacing	1.90~11.29	6.19 ± 1.60
Caudal peduncle length	9.81~43.37	21.57 ± 4.95
Caudal peduncle height	2.12~14.08	6.43 ± 1.82

Note: ^1^ SD represents standard deviation.

**Table 2:** Ratio of measurable characteristics of *T. strauchii*. (*n* = 731; mm).

Indicator	Male		Female	
	Range	Mean ± SD ^1^	Range	Mean ± SD ^1^
Total length/body length	0.38~1.74	1.19 ± 0.05	0.90~2.54	1.19 ± 0.79
Body length/body width	4.71~16.08	8.50 ± 1.39	3.86~15.29	8.58 ± 1.62
Body length/body depth	4.81~13.45	7.30 ± 1.20	3.89~13.11	7.37 ± 1.20
Body depth/body width	0.70~2.09	1.18 ± 0.22	0.59~2.13	1.18 ± 0.22
Body length/head length	3.99~36.59	5.08 ± 1.82	4.01~12.46	5.04 ± 0.88
Body length/caudal peduncle length	2.78~16.36	4.63 ± 0.89	2.09~8.94	4.64 ± 0.67
Body length/eye diameter	17.33~77.40	32.24 ± 7.27	10.53~117.19	32.14 ± 8.64
Body length/eye spacing	7.66~25.37	16.14 ± 2.55	9.85~32.32	16.26 ± 2.80
Head length/snout length	0.30~6.53	2.60 ± 0.70	0.96~8.40	2.68 ± 0.88
Head length/eye diameter	1.07~14.60	6.47 ± 1.49	2.04~23.75	6.48 ± 1.83
Head length/caudal peduncle length	0.16~3.30	0.93 ± 0.20	0.35~2.20	0.94 ± 0.16
Head length/eye spacing	0.49~4.97	3.24 ± 0.58	0.98~6.33	3.28 ± 0.65
Caudal peduncle length/caudal peduncle height	1.11~5.26	3.42 ± 0.64	1.42~6.18	3.46 ± 0.74

Note: ^1^ SD represents standard deviation.

**Table 3:** Table of *T. strauchii* measurable trait ratios’ characteristic values in PC1–PC3. (*n* = 731; mm).

Character	PC1	PC2	PC3
Total length/body length	−0.131	−0.024	0.160
Body length/body width	0.136	−0.076	0.853
Body length/body depth	0.379	−0.224	−0.122
Body depth/body width	−0.195	0.118	0.898
Body length/head length	−0.322	−0.218	−0.048
Body length/caudal peduncle length	0.164	0.854	−0.037
Body length/eye diameter	0.715	−0.223	−0.067
Body length/eye spacing	0.667	−0.124	0.192
Head length/snout length	0.627	0.065	−0.159
Head length/eye diameter	0.82	−0.112	−0.033
Head length/caudal peduncle length	0.323	0.894	0.006
Head length/eye spacing	0.756	0.031	0.203
Caudal peduncle length/caudal peduncle height	0.161	−0.711	0.056

**Table 4:** Truss structure distances of *T. strauchii*. (*n* = 768, mm).

Location	Range	Mean ± SD ^1^
A–B	5.91~26.94	18.13 ± 2.57
B–C	12.97~61.89	35.35 ± 7.83
C–D	3.87~20.61	10.62 ± 2.65
D–E	9.94~69.19	35.62 ± 7.89
E–F	2.28~12.81	6.94 ± 1.85
F–H	5.50~50.63	24.90 ± 6.09
H–I	6.56~42.57	19.00 ± 4.89
I–J	11.82~63.70	33.58 ± 7.61
A–J	7.19~64.00	21.99 ± 5.27
A–C	22.80~76.12	52.21 ± 10.75
A–I	21.81~79.98	54.47 ± 11.09
B–J	4.18~20.71	11.13 ± 2.92
B–I	12.53~59.44	38.50 ± 8.85
C–J	12.90~59.17	33.75 ± 7.54
C–I	2.29~26.09	13.22 ± 3.42
C–H	4.99~45.71	23.05 ± 6.08
C–F	20.05~82.69	45.49 ± 9.74
D–I	4.78~27.37	13.46 ± 3.59
D–H	4.39~27.68	14.49 ± 4.01
D–F	7.68~55.53	35.50 ± 7.72
E–H	11.33~57.78	27.18 ± 6.26

Note: ^1^ SD represents standard deviation. A: tip of snout; B: the last end of the frontal maxilla; C: origin of dorsal fin; D: basal end of the dorsal fin; E: dorsal origin of caudal fin; F: ventral origin of the caudal fin; H: anal fin origin; I: ventral fin origin; J: pectoral fin origin.

**Table 5:** Growth parameters of different *Triplophysa* species. *b*: allometric growth factor; *t_i_*: growth inflection point age; *φ*: growth characteristic index; *k*: growth coefficient.

Fish Species	Data Source	Body Length/mm	Body Weight/g	*b*	*t_i_* (♀)	*t_i_* (♂)	*t_i_*	*φ*	*k*
*T. strauchii*	This study	43.50~142.14	0.60~36.71	3.073	2.304	1.994	2.563	3.668	0.267
*T. stoliczkae*	[64]	27.86~66.43	4.00~60.32	3.234					
*T. yarkandensis*	[65]	38~290	0.80~271.60	2.856	8.50	5.90		4.228	
*T. stewarti*	[32]	34.70~143	0.40~28.70	3.012			3.65		
*T. orientalis*	[31]	49~130	2.10~23.50	2.930	8.19	5.83			0.183
*T. markehenensis*	[47]	34.30~145	34.3~145	2.952			6.25		
*T. tenuis*	[66]	37.02~129.64	0.10~15.15	2.794			2.57		
*T. scleroptera*	[67]	47.80~178.10	0.97~47.20	2.989			10.03		0.097
*T. siluroides*	[68]	20.10~83.90	0.68~5.51	2.699			2.14		

## Data Availability

The datasets presented in this article are not readily available because the data are part of an ongoing study or due to technical/time limitations. Requests to access the datasets should be directed to Zhulan Nie.

## Appendix A

Based on the actual measurement data, the average actual body length of *T. strauchii* in Xinjiang at different age groups was calculated using the nonlinear regression method. The growth relationship between the body length and weight of *T. strauchii* in Xinjiang was fitted with the von Bertalanffy growth equation, and these fitted parameters were input into the von Bertalanffy growth equation to derive the growth equations for the body length and weight of *T. strauchii*. Based on the growth equations, the first-order and second-order derivatives were calculated, thus obtaining the growth rate and growth acceleration equations of these three equations, respectively.

Female population: *L*_∞_ = 142.238; *W*_∞_ = 7.85; *k* = 0.24; *t_0_* = −2.217; *t_i_* = 2.304; *φ* = 3.68.

*L_t_* = 142.238 [1 − *e*^−0.255(*t*+4.598)^]

*W_t_* = 7.85 [1 − *e*^−0.255(*t*+4.598)^]^2.961^

*dL/dt* = 34.137 *e*^−0.255(*t*+4.598)^

*dW/dt* = 5.579 *e*^−0.255(*t*+4.598)^ [1 − *e*^−0.255(*t*+4.598)^]^1.961^

*d*^2^*L*/*dt*^2^ = −8.193 *e*^−(*t*+4.598)^

*d*^2^*W*/*dt*^2^ = 23.30 *e*^−0.255(*t*+4.598)^ [1 − *e*^−0.255(*t*+4.598)^]^0.961^ [2.961 *e*^−0.255(*t*+4.598)^ − 1]

Male population: *L*_∞_ = 129.871; *W*_∞_ = 1.59; *k* = 0.277; *t_0_* = −2.096; *t_i_* = 1.994; *φ* = 3.668.

*L_t_* = 129.871 [1 − *e*^−0.277(*t*+2.096)^]

*W_t_* = 1.59 [1 − *e*^−0.277(*t*+2.096)^]^3.105^

*dL/dt* = 35.974 *e*^−0.277(*t*+2.096)^

*dW/dt* = 1.368 *e*^−0.277(*t*+2.096)^ [1 − *e*^−0.277(*t*+2.096)^]^2.105^

*d*^2^*L*/*dt*^2^ = −9.965 *e*^−(*t*+2.096)^

*d*^2^*W*/*dt*^2^ = 3.788 *e*^−0.277(*t*+2.096)^ [1 − *e*^−0.277(*t*+2.096)^]^1.105^ [3.105 *e*^−0.277(*t*+2.096)^ − 1]

Overall population: *L*_∞_ = 139.46; *W*_∞_ = 27.79; *k* = 0.267; *t_0_* = −1.639; *t_i_* = 2.563; *φ* = 3.715.

*L_t_* = 139.46 [1 − *e*^−0.267(*t*+1.639)^]

*W_t_* = 27.79 [1 − *e*^−0.267(*t*+1.639)^]^3.073^

*dL/dt* = 37.236 e−0.267(t + 1.639)

*dW/dt* = 15.382 *e*^−0.267(*t*+1.639)^ [1 − *e*^−0.267(*t*+1.639)^]^2.073^

*d*^2^*L*/*dt*^2^ = −9.942 *e*^−(*t*+1.639)^

*d*^2^*W*/*dt*^2^ = 6.088 *e*^−0.267(*t*+1.639)^ [1 − e *e*^−0.267(*t*+1.639)^]^1.073^ [3.073 *e*^−0.267(*t*+1.639)^ − 1]
